# Integrated metabolomic and transcriptomic study unveils the gene regulatory mechanisms of sugarcane growth promotion during interaction with an endophytic nitrogen-fixing bacteria

**DOI:** 10.1186/s12870-023-04065-6

**Published:** 2023-01-24

**Authors:** Qian Nong, Mukesh Kumar Malviya, Manoj Kumar Solanki, Li Lin, Jinlan Xie, Zhanghong Mo, Zeping Wang, Xiupeng Song, Xin Huang, Changning Li, Yangrui Li

**Affiliations:** 1 Key Laboratory of Sugarcane Biotechnology and Genetic Improvement (Guangxi), Ministry of Agriculture and Rural Affairs, Guangxi Key Laboratory of Sugarcane Genetic Improvement, Nanning, 530007 China; 2 Plant Protection Research Institute, Guangxi Academy of Agricultural Sciences, Nanning, 530007 China; 3 Plant Cytogenetics and Molecular Biology Group, Institute of Biology, Biotechnology and Environmental Protection, Faculty of Natural Sciences, University of Silesia in Katowice, 40-032 Katowice, Poland

**Keywords:** Nitrogen-fixing bacteria, Sugarcane, Transcriptome, Metabolome, Flavonoid biosynthesis

## Abstract

**Background:**

Sugarcane growth and yield are complex biological processes influenced by endophytic nitrogen-fixing bacteria, for which the molecular mechanisms involved are largely unknown. In this study, integrated metabolomic and RNA-seq were conducted to investigate the interaction between an endophytic bacterial strain, *Burkholderia* GXS16, and sugarcane tissue culture seedlings.

**Results:**

During treatment, the colonization of GXS16 in sugarcane roots were determined, along with the enhanced activities of various antioxidant enzymes. Accordingly, 161, 113, and 37 differentially accumulated metabolites (DAMs) were found in the pairwise comparisons of adjacent stages. In addition, transcriptomic analyses obtained 1,371 (IN-vs-CN), 1,457 (KN-vs-IN), and 365 (LN-vs-KN) differentially expressed genes (DEGs), which were mainly involved in the pathways of glutathione metabolism and carbon metabolism. We then assessed the pattern of metabolite accumulation and gene expression in sugarcane during GXS16 colonization. The results showed that both DAMs and DGEs in the upregulated expression profiles were involved in the flavonoid biosynthesis pathway. Overall, p-coumaroyl-CoA in sugarcane roots transferred into homoeriodictyol chalcone and 5-deoxyleucopelargonidin due to the upregulation of the expression of genes shikimate O-hydroxycinnamoyltransferase (HCT), chalcone synthase (CHS), and phlorizin synthase (PGT1).

**Conclusions:**

This study provides insights into the gene regulatory mechanisms involved in the interaction between GXS16 and sugarcane roots, which will facilitate future applications of endophytic nitrogen-fixing bacteria to promote crop growth.

**Supplementary Information:**

The online version contains supplementary material available at 10.1186/s12870-023-04065-6.

## Background

Nitrogen (N) is a key component of DNA, RNA, proteins, and various nitrogenous metabolites, and plays an essential role in cell development and life activity. Normally, soil N is available for plant nutrition, and its input largely depends on organic matter degradation, mineral fertilizer application, and biological nitrogen fixation (BNF) via plant-associated microorganisms. To alleviate N limitation and maximize crop productivity, copious amounts of nitrogenous chemical fertilizers are produced, and more than 90% of produced N fertilizers are applied in human agricultural practice (approximately 60% for cereals and 10% for irrigated rice) [1] however, this practice comes at high environmental costs, such as greenhouse gas emissions, nitrate contamination of soil and ground water, and damage to agricultural sustainability, thus threatening human health [2, 3]. In contrast, the process of BNF, involving many physiological and biochemical modifications in crop tissues, can convert N_2_ into a plant-usable form (inorganic nitrogen-containing compounds), such as ammonia (NH_3_), and contributes to the growth, development, fitness, and diversification of sorghum [4], rice [5], cucumber [6], wheat [6], cassava [7], switchgrass [8], maize [9], and other plants in arable lands and natural ecosystems. Thus, BNF has gathered attention for promoting crop growth and achieving agro-environmental sustainability worldwide.

Nitrogen-fixing microorganisms usually encode nitrogenase which is regulated by *nifH*, *nifD*, *nifK*, *nifE*, *nifN*, *nifX*, *nifQ*, *nifW*, *nifV*, *nifA*, *nifB*, *nifZ*, and *nifS,* and contributed to BNF [10, 11]. These organisms, including gram-negative and gram-positive bacteria, are involved in various endosymbiotic interactions with host plants. A previous study showed that the effective use of bioinoculants containing nitrogen-fixing microorganisms can reduce the use of nitrogenous fertilizers. *Paenibacillus beijingensis* BJ-18 can provide 12.9–20.9% N to wheat and 52.2–59.2% to cucumber through BNF. Simultaneously, inoculation significantly increased shoot dry weight (wheat 86.1%, maize 46.6%, and cucumber 103.6%) and root dry weight (wheat 46.0%, maize 47.5%, and cucumber 20.3%) [12]. *Burkholderia vietnamiensis* (MGK3 and LMG10929), *Gluconacetobacter diazotrophicu*s, *Herbaspirillum seropedicae*, and *Azospirillum lipoferum* provided 12–33% total N, and combined inoculation increased rice yield by 9.5–23.6%, whereas MGK3 alone increased yield by 5.6–12.16% than the uninoculated control treatment [5]. In addition to BNF, associated nitrogen-fixing microorganisms interact with plants in many pathways to increase crop resistance to plant-associated pathogens, insects, and abiotic stress. Many nitrogen-fixing microorganisms can manipulate metabolism of plant hormone, such as auxins, cytokinins, gibberellins, abscisic acid, ethylene, salicylic acid, jasmonates, and brassinosteroids, to promote the benefits for both partners in symbiosis.

Sucrose, contributed by sugarcane, accounts for 90% of global production. Guangxi produces more than 60% of China’s sugarcane and sugar, making it the third largest sugar producer in the world, after Brazil and India [13]. However, most farmers in China have a misconception that more N fertilizer will contribute to higher sugarcane growth; this has led to overfertilization which causes low fertilizer utilization efficiency. For example, the utilization of chemical fertilizers such as nitrogen (urea) in China ranged from to 400–800 kg ha^−1^ year^−1^, which is considerably higher than those utilized in Australia, Brazil, and India (60–100, 150–400, and 160–200 kg ha^−1^ year^−1^, respectively) [14]. The accumulated data revealed the vital and novel results of many uncharted nitrogen-fixing microorganisms in different sugarcane tissues, such as *Azospirillum* [15], *Klebsiella variicola* [16], *Pseudomonas koreensis* [17], and *Pseudomonas entomophila* [17], which provide an experimental system to evaluate BNF mechanisms in sugarcane. The interaction between nitrogen-fixing microorganisms and sugarcane should be exploited to decrease the use of chemical fertilizers in sugarcane production, thereby decreasing production costs and improving fertilizer-use efficiency while ensuring high cane and sugar productivity.

*Burkholderia* GXS16, an endophytic nitrogen-fixing bacterial strain, has been shown to efficiently fix N_2_ in sugarcane and promote sugarcane growth in our previous study [18]. To investigate the regulatory mechanism of this bacterial strain on sugarcane tissue culture seedlings, we performed biochemical characterization, metabolomic profiling, and transcriptomic analysis in the present study. Differentially accumulated metabolites (DAMs) and differentially expressed genes (DEGs) during GXS16 inoculation in sugarcane roots were identified, and their changing trends were analyzed to reveal key regulatory pathways. The data from this study provides insights into the gene regulatory mechanisms of sugarcane growth promotion during interaction with the endophytic nitrogen-fixing bacteria.

## Results

### Enumeration of GXS16 and biochemical characterization in sugarcane root

**Fig. 1:**
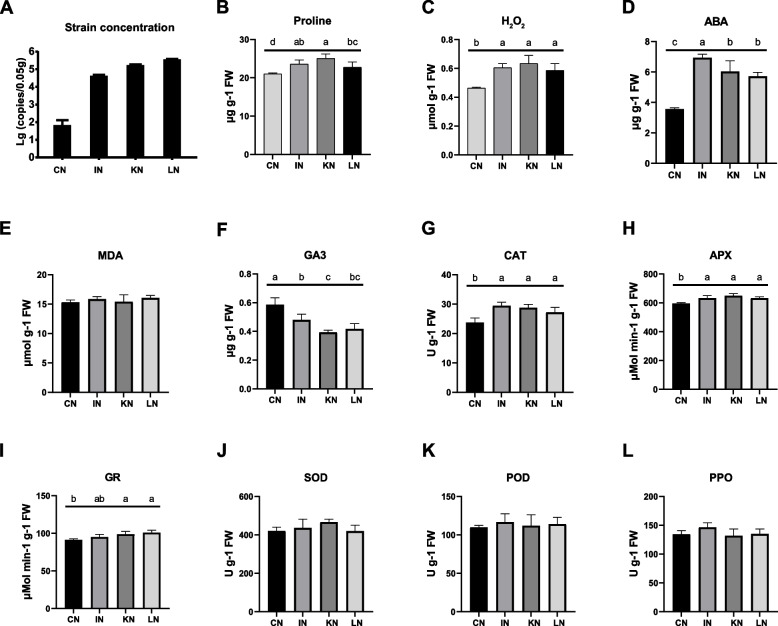
**A** Bacterial density of GXS16 in sugarcane root at four different stages (CN: 0 h, IN: 6 h, KN: 12 h and LN: 24 h). The changes in the biochemical indices of sugarcane roots at four different stages during GXS16 incubation. **B** Proline content. **C** H_2_O_2_ content. **D** Abscisic acid (ABA) content. **E** Malondialdehyde (MDA) content. **F** Gibberellin (GA) content. **G** Catalase (CAT) activity. **H** Ascorbate peroxidase (APX) activity. **I** Glutathione reductase (GR) activity. **J** Superoxide dismutase (SOD) activity. **K** Peroxidase (POD) activity. **L** Polyphenol oxidase (PPO) activity

After incubation for 6 h, the density of GXS16 in *Saccharum officinarum* root increased by 2.80 ± 0.26 lg copies/0.05 g (Fig. 1A). At 12 h and 24 h post-incubation, the increase in bacterial colonization in sugarcane roots slowed down. We then assessed the changes in the biochemical indices of sugarcane roots during GXS16 incubation. Accordingly, the content of proline, H_2_O_2_, and endogenous abscisic acid (ABA, Fig. 1B–D) was significantly elevated from 0 h to 6 h post-incubation (ANOVA, *P* < 0.05). In contrast, malondialdehyde (MDA) content did not significantly change during GXS16 incubation (Fig. 1E), and GXS16 colonization significantly decreased gibberellin (GA) content in sugarcane roots (Fig. 1F). In addition, GXS16 colonization significantly elevated the activities of catalase (CAT), ascorbate peroxidase (APX), and glutathione reductase (GR) in sugarcane roots (ANOVA, *P* < 0.05, Fig. 1G-I), but had no significant impact on the activities of superoxide dismutase (SOD), peroxidase (POD), and polyphenol oxidase (PPO, Fig. 1J-L)

### Metabolomic changes in sugarcane root during GXS16 inoculation

**Fig. 2:**
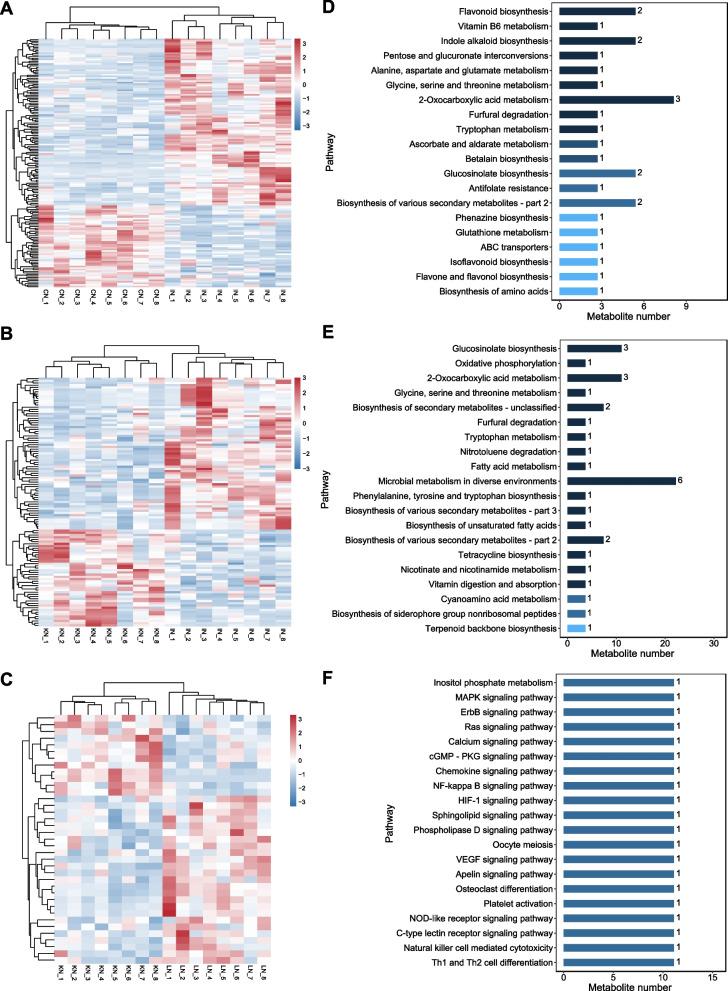
Metabolomic changes in sugarcane root during GXS16 inoculation (CN: 0 h, IN: 6 h, KN: 12 h and LN: 24 h). Heatmaps showing the differentially accumulated metabolites in the comparison of IN vs CN (**A**), KN vs IN (**B**), and LN vs KN (**C**). The KEGG pathway annotations from DAMs in IN vs CN (D), KN vs IN (**E**), and LN vs KN (**F**) are shown

Metabolomic analyses identified 3,540 metabolites (Additional Table S1). Among these metabolites, 108 and 53 were significantly high- and low-abundant in the comparison to that of IN-vs-CN (the comparison between 6 h and 0 h post-incubation, Fig. 2A, Additional Table S2), 44 and 69 were significantly high- and low-abundant in the comparison to that of KN-vs-IN (the comparison between 12 h and 6 h post-incubation, Fig. 2B, Additional Table S3), and 25 and 12 were significantly high- and low-abundant in the comparison to that of LN-vs-KN (the comparison between 24 h and 12 h post-incubation, Fig. 2C, Additional Table S4). KEGG pathway annotation was performed on the DAMs in the three comparisons. The DAMs in IN-vs-CN were largely involved in the pathways of flavonoid biosynthesis, indole alkaloid biosynthesis, and 2-oxocarboxylic acid metabolism (Fig. 2D), and mainly participated in the pathways of glucosinolate biosynthesis, 2-oxocarboxylic acid metabolism, and biosynthesis of secondary metabolites (Fig. 2E). Various signaling pathways were annotated for DAMs in LN-vs-KN (Fig. 2F).

### Transcriptomic changes in sugarcane root during GXS16 inoculation

**Fig. 3:**
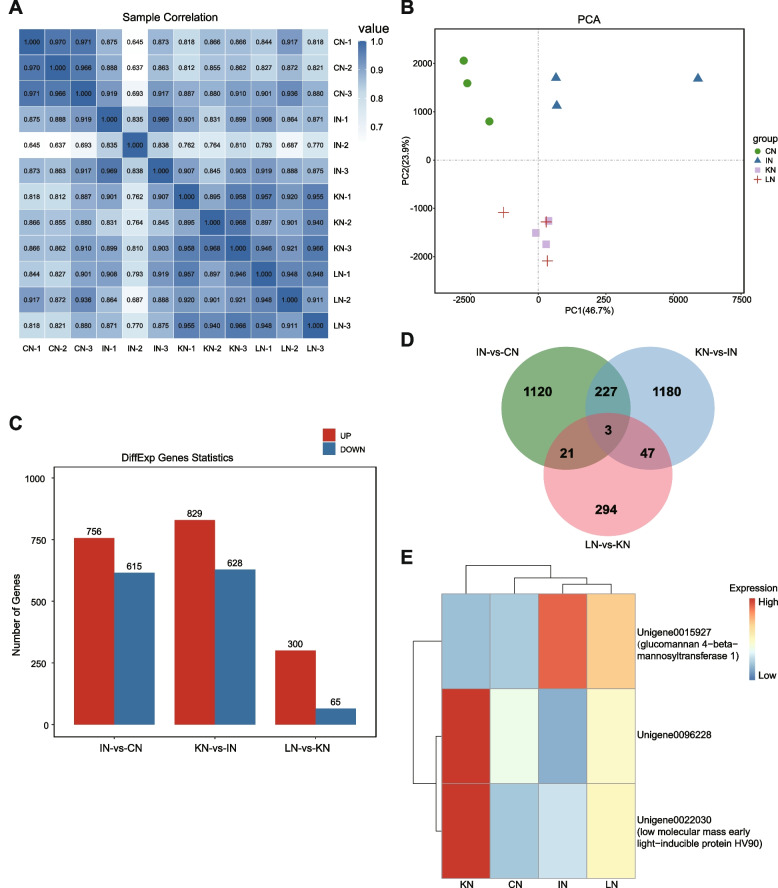
Differential gene expressions in sugarcane root during GXS16 inoculation. **A** Thermal diagram of the correlation coefficient among RNA-seq samples. **B** Principal component analysis of RNA-seq samples. **C** Statistics of DEGs in different comparisons (IN vs CN, KN vs IN, and LN vs KN). **D** Venn diagram showing the overlap of DEGs among different comparisons. (E) Heatmap showing the expression level of three overlapped DEGs

RNA-seq was performed to identify gene regulatory mechanisms related to metabolic changes during the interaction between GXS16 and sugarcane roots. The sequencing produced a total of 131.6 Gb of clean data, with an average of 10.9 Gb for each sample. More than 88% of sequenced bases have a quality score of Q30 or higher, indicating high quality sequencing data for downstream analysis (Additional Table S5). After assembly, 101,097 unigenes with an average length of 957 bp, were obtained (Additional Table S6). The BUSCO assessment results of assembly showed that more than 76% of the BUSCOs were complete, which suggested the high completeness of assembled unigenes (Additional Fig. S1). The unigenes were annotated using multiple databases, and 49,172 unigenes were annotated using at least one database (Additional Table S6). The Pearson’s correlation between replicates ranged from 0.84 to 0.97, suggesting that the transcriptome results were reliable and stable (Fig. 3A). PCA analysis indicated that all the inoculated samples were clustered apart from the initial samples (CN), and the KN and LN samples were clustered together (Fig. 3B). Accordingly, 1,371, 1,457, and 365 DEGs were identified in the pairwise comparisons of IN-vs-CN, KN-vs-IN, and LN-vs-KN, respectively (Fig. 3C, Table S7-9). Among these DEGs, three genes were differentially expressed in all comparisons (Fig. 3D). The expression of Unigene0015927 encoding glucomannan 4−beta−mannosyltransferase 1 was upregulated, whereas the expressions of Unigene0096228 without annotation and Unigene0022030 encoding a low molecular mass early light-inducible protein were downregulated after GXS16 colonization (Fig. 3E).

KEGG pathway enrichment analyses revealed that the DEGs in IN-vs-CN were mainly involved in flavonoid biosynthesis, phenylpropanoid biosynthesis, and glutathione metabolism (Additional Fig. S2). The DEGs in KN-vs-IN were largely enriched in glutathione metabolism, carotenoid biosynthesis, and pentose phosphate pathways (Additional Fig. S2). The DEGs in LN-vs-KN were significantly enriched in the DNA replication, mismatch repair, nucleotide excision repair, homologous recombination, base excision repair, pyrimidine metabolism, and starch and sucrose metabolism pathways (Additional Fig. S2). Notably, the DEGs enriched in glutathione metabolism pathway included multiple up-regulated antioxidative genes, such as GSTU6 (Unigene0061796, Unigene0089252, Unigene0063190, Unigene0005938, Unigene0002117, Unigene0061797, Unigene0061798 and Unigene0087068), HSP26-A (Unigene0052944), G6PDH (Unigene0096221), GST4 (Unigene0026305), and GSTU17 (Unigene0063939) in IN-vs-CN (Table S7), APX2 (Unigene0021368) in KN-vs-IN (Table S8), and GSTF11 (Unigene0096075) in LN-vs-KN (Table S9).

### Differential trends of metabolites and genes in sugarcane root during GXS16 inoculation

**Fig. 4:**
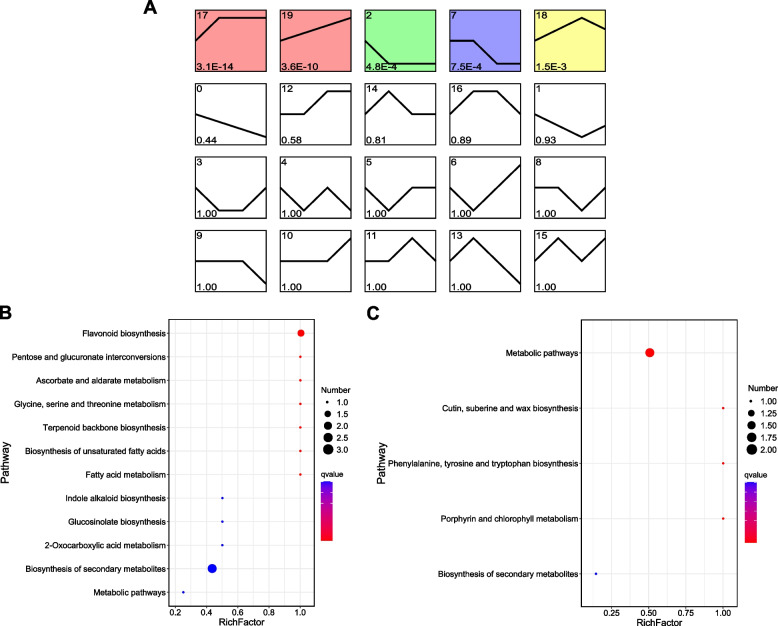
Temporal trends of metabolite accumulation in sugarcane root during GXS16 inoculation. **A** STEM analysis of the DAMs. Each box indicates a model profile, and the colored profiles shown are significant. Profile numbers are indicated in the top left-hand corner, and the corresponding *P*-values for each profile are shown in the bottom left-hand corner. KEGG pathway enrichment of significant upregulated (**B**) and downregulated (**C**) profiles are shown

To investigate the pattern of metabolite accumulation, the 110 DAMs were clustered using STEM software, and the results showed that the changing trends of most DAMs were classified into profiles 17, 19, 2, 7, and 18 (*P* < 0.05, Fig. 4A). Among the significantly clustered profiles, profiles 17 and 19 contained metabolites whose abundance showed upregulated trends across stages, and profiles 2 and 7 contained metabolites whose abundance was downregulated across stages. The metabolites in the upregulated profiles were involved in flavonoid biosynthesis, terpenoid backbone biosynthesis, and fatty acid metabolism (Fig. 4B), while metabolites in the downregulated profile participated in the pathways of porphyrin and chlorophyll metabolism, cutin, suberin, and wax biosynthesis, and phenylalanine, tyrosine, and tryptophan biosynthesis (Fig. 4C).

**Fig. 5:**
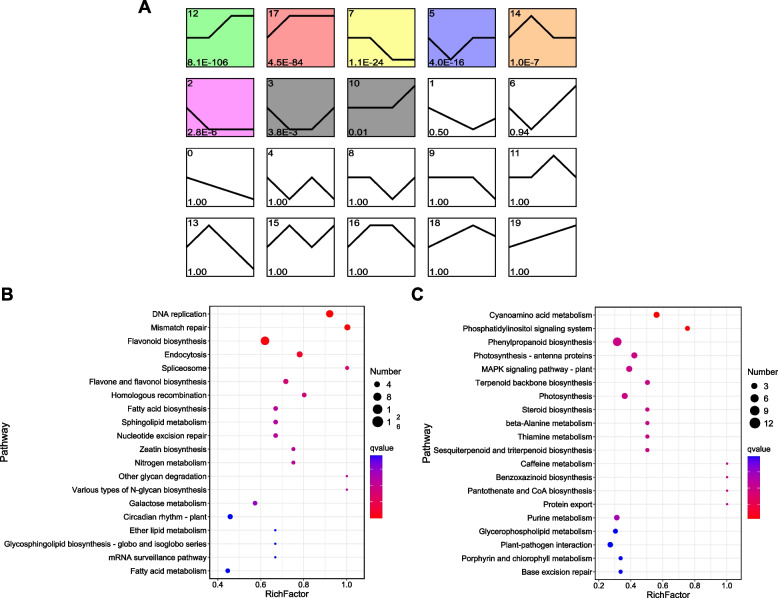
Temporal trends of gene expression in sugarcane root during GXS16 inoculation. **A** STEM analysis of the DEGs. Each box indicates a model profile, and the colored profiles shown are significant. Profile numbers are indicated in the top left-hand corner, and the corresponding *P*-values for each profile are shown in the bottom left-hand corner. KEGG pathway enrichment of significant upregulated (**B**) and downregulated (**C**) profiles are shown

STEM was also used to estimate gene expression trends during GXS16 inoculation. The expression of 2,892 DEGs were clustered into 20 profiles, of which profiles 12, 17, 7, 5, 14, 2, 3, and 10 were significantly clustered by genes (Fig. 5A). Among them, profiles 10, 12, and 17 contained genes whose expression showed upregulated trends across stages, and profiles 2 and 7 contained genes whose expression was downregulated across stages. The genes in the upregulated profile were enriched in the pathways of DNA replication, mismatch repair, flavonoid biosynthesis, and endocytosis (Fig. 5B), while genes in the downregulated profiles were mainly involved in the pathways of cyanoamino acid metabolism and the phosphatidylinositol signaling system (Fig. 5C).

**Fig. 6:**
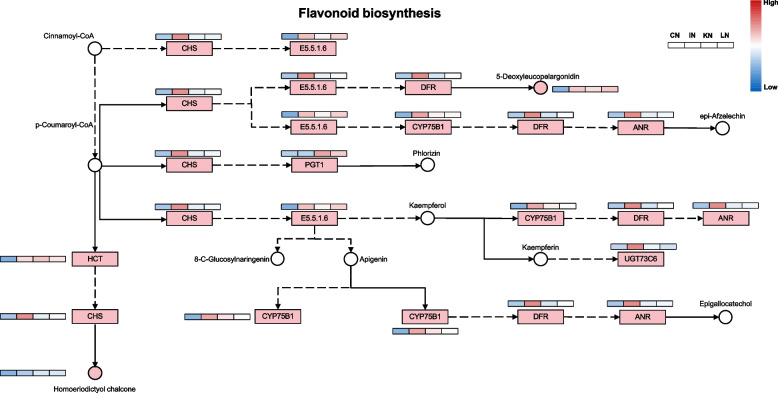
Changes in gene expression and metabolite accumulation in the pathway of flavonoid biosynthesis [19]. Metabolites and genes marked with red indicate significantly upregulated in sugarcane root. The heatmap represents the accumulation or expression level of corresponding factor in four stages

**Fig. 7:**
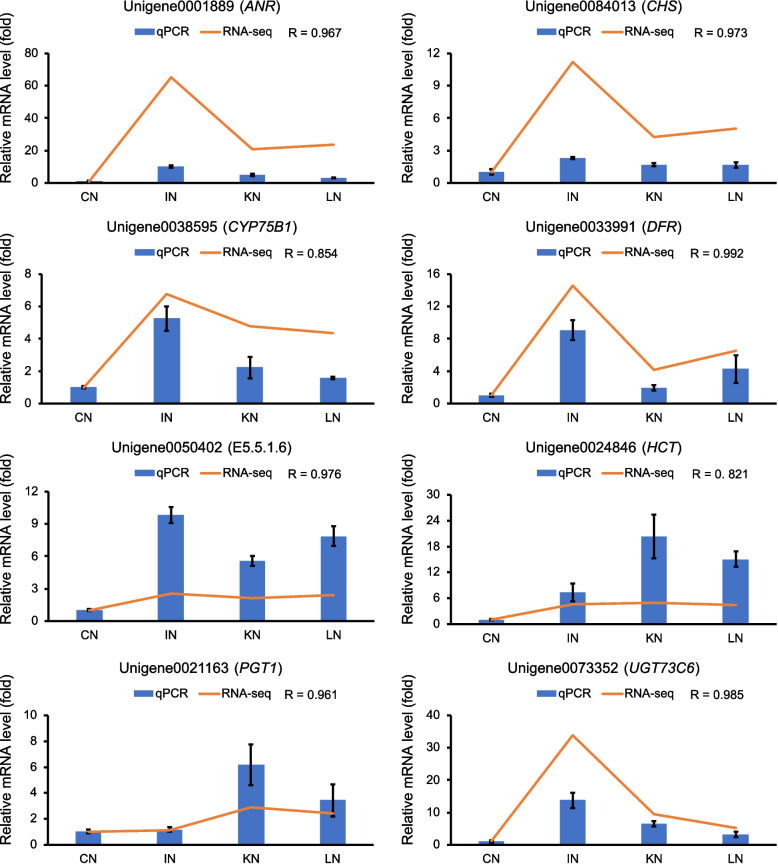
qRT-PCR validation on the mRNA level of the key genes involved in the the flavonoid biosynthesis pathway. Relative mRNA levels of qRT-PCR were calculated using GAPDH as an internal control. R value in the top right of each figure indicates Pearson correlation coefficient between relative mRNA levels from qRT-PCR and transcriptome across stages

Notably, the flavonoid biosynthesis pathway was affected by both the upregulated DAMs and DEGs. Accordingly, the metabolic changes in this pathway were constructed (Fig. 6). During GXS16 inoculation, p-coumaroyl-CoA in sugarcane roots transferred into homoeriodictyol chalcone and 5-deoxyleucopelargonidin due to the upregulation of expression of multiple genes in the flavonoid biosynthesis pathway such as shikimate O-hydroxycinnamoyltransferase (HCT), chalcone synthase (CHS), and phlorizin synthase (PGT1). The expression of all key genes in this pathway was further confirmed by quantitative real-time PCR (qRT-PCR), and the results showed high consistency between transcriptome and qRT-PCR (Fig. 7).

## Discussion

Compared with other tissues and organs in sugarcane, the roots offer more carbohydrates for the function of nitrogenase, and the partial pressure of oxygen in the surroundings of roots is lower, which have an essential role in preventing the oxidation of nitrogenase. Therefore, it contributes to the BNF of sugarcane. In this study, the concentration of GXS16 was evident throughout the experiments, as shown by a significant increase in colonization after 6 h of inoculation. In the previous study, images taken using a fluorescent confocal microscope indicated that the tagged GXS16 penetrate the root surface and elongation zones 24 h after inoculation and carry increased nitrogenase activity [20]. We also validated that GXS16 increased plant height and dry weight by more than 15% and 20%, respectively, compared to the negative controls. The ^15^N isotope dilution assays demonstrated that the associative nitrogen fixation rates of GXS16 in sugarcane roots, stems, and leaves were 7.69%, 15.64%, and 8.72%, respectively, which were higher than those of the model strain *G. diazotrophicus* PAL5 [21]. The ability of GXS16 to colonize sugarcane plants and function as an effective plant growth-promoting bacteria (PGPB) was demonstrated by the enhancement of BNF.

Reactive oxygen species (ROS) can be produced under normal development, biotic stress, and abiotic stress conditions (salinity, drought, heavy metals), and can destroy critical cellular organelles and cellular membranes by inducing the degradation of pigments, proteins, lipids, and nucleic acids, which cause cell death in plants. However, ROS can be minimized to protect the cell from oxidative damage and coordinate plant growth by a complex battery of enzymatic antioxidative defense systems (H_2_O_2_, CAT, APX, and GR) [22]. In this study, not only higher activities of them were observed, we also detected the upregulated expression of GSTU6, GSTF11, HSP26-A, G6PDH, GST4, GSTU17, and APX2 (Table S7-9), which mainly contributed to metabolic pathways and glutathione metabolism. All these DEGs play a central role in the ascorbate-glutathione pathway and combat the harmful effects of ROS. Thus, GXS16 might tolerate adverse environmental conditions for extended periods and possess biocontrol properties against stress.

Based on metabolomic analysis, several downregulated and upregulated pathways (biosynthesis of secondary metabolites, 2-oxocarboxylic acid metabolism, flavonoid biosynthesis, indole alkaloid biosynthesis, and terpenoid backbone biosynthesis) related to the sugarcane tolerance phenotype were obtained. Here, we discuss and highlight the biosynthesis of secondary metabolites. Secondary metabolites mainly consist of nitrogen-containing molecules (alkaloids) and nitrogen-deficient molecules (terpenoids). Alkaloids, including terpenoid indole alkaloids (e.g., vinblastine and vincristine), tropane alkaloids (cocaine, scopolamine), and purine alkaloids (caffeine), are nitrogen-containing molecules that are known to protect plants from microbial or herbivore attack and even carry high pharmaceutical value for the treatment of terminal diseases [23–25]. Terpenoids, synthesized either in the cytosol (mevalonate pathway) or in chloroplasts (methylerythritol phosphate pathway), are derived from the universal five-carbon precursors isopentenyl diphosphate and dimethylallyl diphosphate [23]. Plants have terpenoid-related defense systems when they are attacked by herbivores or infected by fungal and bacterial pathogens [26]. In the present study, the levels of trans-polycis-polyprenyl diphosphate, indolylmethyl-desulfoglucosinolate, and 3-methylthiopropyl-desulfoglucosinolate continued to increase within 12 h of inoculation with GXS16. These results indicate that they are beneficial for the defense responses of plants to environmental stressors.

Flavonoids, comprising a C15 benzene ring structure of C6–C3–C6, are widely distributed secondary metabolites and are derived from the phenylpropanoid metabolic pathway. Transcriptomic and metabolomic analyses revealed that flavonoid biosynthesis, especially the biosynthesis of 5-deoxyleucopelargonidin and homoeriodictyol chalcone, was upregulated and several DEGs were involved in this pathway. For example, CHS (the first enzyme in the pathway branch for flavonoid biosynthesis) was upregulated in IN, indicating that GXS16 in sugarcane causes an increase in CHS transcript abundance. A previous report revealed that CHS genes were significantly upregulated in sugarcane FN41 and 165204, demonstrating that CHS contributes to the synthesis of anthocyanin and lignin and impacts rind color [27]. In addition, the flux of flavonoids contributes to the branch pathway for flavonols, which are suggested to act as signaling molecules that regulate plant growth and development. For example, they can function as endogenous polar auxin transport inhibitors to regulate shoot and root growth under various conditions [28, 29]. However, flavonols are proposed to act as positional signals that integrate hormonal and ROS pathways to regulate the direction and rate of root growth in root negative phototropism [30].

## Conclusions

During the colonization of GXS16, the contents or activities of H_2_O_2_, ABA, APX, proline, CAT, and GR increased in sugarcane roots, which might protect sugarcane from pathogens. Based on metabolomic and transcriptomic analyses, we identified various DAMs and DEGs potentially associated with sugarcane growths. In particular, the upregulation of the flavonoid biosynthesis pathway in sugarcane might provide a supplement and target for the application of endophytic nitrogen-fixing bacteria in sugarcane.

## Methods

### Material information and field conditions

*Burkholderia* endophytic nitrogen-fixing bacteria GXS16 were isolated and purified from Guitang 31 by our research team. The pGXS16 was stored in molecular grade water at –20 °C. Plant materials of sugarcane variety RB86-7515 were provided by the Sugarcane Research Institute of Guangxi Academy of Agricultural Sciences (Guangxi Zhuang Autonomous Region, China). We got the permission to collect the plant materials.

The rooted clump sugarcane tissue culture seedlings were divided into individual plants, transferred to culture flasks (containing 1/10 MS liquid medium, without vitamins and plant hormones), and maintained at 30°C, 16 h light/8 h dark, and 60 μmol photons m^-2^ s^-1^ in a light incubator (GXM-808，Ningbo Jiangnan Instrument Co.,Ltd., China) [21]. After seven days, plants with the similar growth and development were selected and moved into flower pots filled with a fully sterilized mixture of sand and perlite (v/v = 1:1) and placed into the artificial climate box (RXZ-1500，Ningbo Jiangnan Instrument Co.,Ltd., China) under the condition of 30°C, 16 h light/8 h dark, and 120 μmol photons m^-2^ s^-1^. Plants were watered with 200 mL of 1/10 MS nutrient solution every three days as needed (200 mL of sterile water per day at other times). GXS16 growing in log phase was collected by centrifugation (4,000 g, 10 min, 25 °C), washed twice with 1/10 MS medium, and diluted to obtain a bacterial suspension of 1–2 × 10^8^ CFU/mL. Then, all roots in the infected group were injected with 300 mL of GXS16 suspension, whereas those in the control group were injected with the same volume of sterile water. Root samples were collected at 0 h (CN), 6 h (IN), 12 h (KN), and 24 h (LN) after inoculation. All samples were aliquoted and snap-frozen in liquid nitrogen prior to further experiments.

### Detection of the copy number of colonized GXS16 in sugarcane root

Whole DNA was extracted from 200 mg of sugarcane root using the cetyltrimethylammonium bromide (CTBA) method according to the methods of a previous report with some modifications [31]. The copy number of GXS16 was determined by quantitative real-time PCR (qRT-PCR) using an ABI 7500 qRT-PCR platform (Applied Biosystems, Foster City, CA, USA). First, specific primers (F:5’-GCAGGCGGTTTGCTAAGACC-3’, R:5’-GCTTTCGTGCATGAGCGTCA-3’, probe:5’-CGGGCTCAACCTGGGAACTGC-3’) were designed and validated using Oligo software (v7.0) and synthesized by Sangon Biotech (Shanghai, China). Second, each qRT-PCR reaction was added to 5 μL gDNA, 10 μL of 2×SYBR Green I Master Mix (Takara, Kyoto, Japan), 0.5 μL F/R primer (10 μM), 0.5 μL probe, and 3.5 μL ddH2O, under qRT-PCR conditions of 30 s at 95°C; 40 cycles of 5 s at 95°C and 30 s at 60°C. All qRT-PCR assays were performed in triplicate. After the qRT-PCR assays, the copy number of GXS16 in root samples was calculated according to the standard curve generated with the plasmid containing the GXS16 sequence.

### Biochemical characterization of sugarcane root

The content of proline, MDA, and H_2_O_2_ in the roots was measured according to previously published methods [32–34]. The contents of ABA and GA were measured using the method described by Iriti et al. [35]. The activities of CAT, POD, PPO, SOD, APX, and GR were measured according to the manufacturer’s instructions using the ELISA kit provided by Keming Biotechnology Co., LTD (Suzhou, China). Three replicates were used for each index.

### Metabolites extraction and LC-MS analysis

Eight replicates of freeze-dried root samples collected at each time point were ground using a mixer mill (MM 400, Retsch, Germany) at 30 Hz for 1.5 min for metabolomic analysis. Approximately 100 mg of powder from each sample was suspended in 1.0 mL of aqueous methanol containing 0.1 mg/L lidocaine as an internal standard and incubated overnight. Then, the mixture was centrifuged (12,000 g, 2 min, 25 °C) and filtered to collect the supernatant. The sample extracts were analyzed using an LC-ESI-MS/MS system (QTRAP 6500; Sciex, USA). The chromatographic separations were performed using a Waters ACQUITY C18 column (2.1 mm × 100 mm, 18 μm, Waters, USA) at a flow rate of 0.4 mL/min at 40°C. Mobile phase A consisted of water (0.04% acetic acid), while phase B consisted of acetonitrile (0.04% acetic acid). The separation was run under the following gradient conditions: 95:5 phase A/phase B for the first 10 min, 5:95 phase A/phase B for the 11th to 12th min, and 95:5 phase A/phase B for the 13th to 15th min. The effluent was detected using a Sciex Triple Quad 6500 mass spectrometer (QTRAP 6500, Sciex, USA) in the positive ion mode.

Metabolite quantification was performed using multiple reaction-monitoring methods. Analyst 1.6.1 software was used for data filtration, peak detection, alignment, and calculation. Metabolites were identified by searching internal and public databases (MassBank, KNApSAcK, HMDB, MoTo Database, and METLIN) with the m/z values, retention times, and fragment patterns. To identify DAMs between pairwise comparisons, we implemented orthogonal partial least squares discriminant analysis (OPLS-DA) using the MetaboAnalystR (1.0.1) package in R according to a threshold: *P*-value ≤ 0.05 (significant difference), variable importance in projection (VIP) ≥ 1, and absolute log2 fold change ≥ 1 [36].

### RNA extraction and RNA-seq analysis

Total RNA was extracted from sugarcane roots using an RNeasy Mini Kit (Qiagen, Hilden, Germany) according to the manufacturer’s instructions. Three replicate samples were extracted from each group. Following extraction, RNA was quantified using a NanoDrop 2000 (Thermo Scientific, Delaware, USA) and Agilent 2100 Bioanalyzer system (Agilent Technologies, Santa Clara, CA, USA). DNase I (Takara, Kyoto, Japan) was used to remove the DNA from the RNA samples. mRNA was enriched using oligo (dT) magnetic beads and converted into short fragments with the addition of fragment buffer. First-strand cDNA was synthesized using a random hexamer primer and mRNA fragments as templates. The second strand was synthesized by adding the buffer, dNTPs, RNase H, and DNA polymerase I. After the double-stranded cDNA was purified, the DNA fragments were subjected to end-repair, base A addition, and sequencing adapter ligation. The fragments were purified using an E-Gel® SizeSelect agarose gel and enriched using PCR amplification. Pair-end reads of 100 bp were produced using the BGISEQ-500 platform (BGI Group, Shenzhen, China) and used for subsequent bioinformatics analysis.

Clean reads were assembled de novo using the Trinity software package (v2.6.6) with default parameters [37]. The assembly was then clustered using the Tgicl software (v2.1) to remove redundancy to obtain unigenes [38]. The completeness of unigenes was assessed using the BUSCO software (v3) with default parameters [39]. The unigenes were BLASTed to databases including nr protein, Swiss-Prot, KEGG [19], Trembl, Gene Ontology (GO), and Clusters of Orthologous Groups of proteins (COG) to obtain functional annotation. To estimate the expression level of unigenes, clean reads were aligned to all transcripts using Bowtie2, and the quantity of gene expression was calculated using RSEM software (v1.2.19) with default parameters [39]. The expression value of each unigene was normalized to fragments per kilobase of transcript per million (FPKM) and transcripts per kilobase million (TPM) fragment mapped reads. DEGs were identified using the DEseq2 package (1.22.2) in R to analyze unstandardized read count data between the two groups gbased on the threshold of false discovery rate (FDR) < 0.05, and absolute log_2_ FC ≥ 1 [40]. Pathway enrichment analysis of the identified metabolites was performed by mapping them to the KEGG compound database. The significant pathways of DAMs were determined using hypergeometric test *P*-values.

### Temporal analysis

Short Time-Series Expression Miner (STEM) software was used to analyze the patterns of DAMs and DEGs in sugarcane roots during the colonization of GXS16 [41]. DAMs and DEGs were clustered according to their *p*-values. Clustered profiles with *P* ≤ 0.05 were considered differentially accumulated or expressed. Metabolites and genes within the selected clusters were enriched in KEGG pathways using a hypergeometric distribution test.

### qRT-PCR analysis

qRT-PCR was conducted to comfirm the expression of key genes. Total RNA was extracted as described above and converted to cDNA using RT SuperMix for qPCR (APEXbio, Houston, USA). Specifc primers of key genes were designed using Primer Premier software (5.0) (Table S10). qRT-PCR was performed using the Bio-Rad iQ5 Real-Time PCR detection system (CA, USA). GAPDH was used as internal control for gene expression normalization and the 2 ^(− ΔCt)^ method was applied to estimate the gene expression values.

### Statistical analysis

Differences among biochemical indices were calculated using one-way analysis of variance (ANOVA) followed by Duncan’s multiple range test. Pearson correlation coeffcient between gene expression values of qRT-PCR and transcriptome was calculated in R v3.6.3. Statistical significance was set at *P* < 0.05.

## Supplementary Information

**Additional file 1: Table S1.** Information of all metabolites identified in this study. **Table S2.** Information of differentially abundant metabolites in IN-vs-CN. **Table S3.** Information of differentially abundant metabolites in KN-vs-IN. **Table S5.** Statistics of RNA-seq data. **Table S6.** Statistics of transcriptome assembly and unigene annotation. **Table S7.** Information of differentially expression genes in IN-vs-CN. **Table S8.** Information of differentially expression genes in KN-vs-IN. **Table S9.** Information of differentially expression genes in LN-vs-KN. **Table S10.** The primers used for qRT-PCR.

**Additional file 2: Fig. S1.** BUSCO assessment of unigene assembly. **Fig. S2.** The root samples were respectively collected at 0 h (CN), 6 h (IN), 12 h (KN) and 24 h (LN) after GXS16 inoculation, and the KEGG pathway enrichment of DEGs in the comparison of IN vs CN, KN vs IN, and LN vs KN.

## Data Availability

The transcriptomic data generated during the current study are available in the NCBI SRA repository under Bioproject No. PRJNA794948, and metabolomic data are deposited to Figshare Dataset (10.6084/m9.figshare.21098884.v2).

## Abbreviations

ANOVA: One-way analysis of variance
DAMs: Differentially accumulated metabolites
DEGs: Differentially expressed genes
HCT: O-hydroxycinnamoyltransferase
CHS: Chalcone synthase
PGT1: Phlorizin synthase
BNF: Biological nitrogen fixation
PGPB: Plant growth-promoting bacteria
ROS: Reactive oxygen species
CTBA: Cetyltrimethylammonium bromide
qRT-PCR: Quantitative real-time PCR
MDA: Malondialdehyde
ABA: Abscisic acid
GA: Gibberellin
CAT: Catalase
POD: Peroxidase
PPO: Polyphenol oxidase
SOD: Superoxide dismutase
APX: Ascorbate peroxidase
GR: Glutathione reductase
FDR: False discovery rate
STEM: Short Time-Series Expression Miner
FPKM: Fragments per kilobase of transcript per million
TPM: Transcripts per kilobase million
