# Supplementary material for: Integrated metabolomic and transcriptomic study unveils the gene regulatory mechanisms of sugarcane growth promotion during interaction with an endophytic nitrogen-fixing bacteria
Source: BMC Plant Biol. 2023 Jan 24;23:54. doi: 10.1186/s12870-023-04065-6 (PMC9872334; doi:10.1186/s12870-023-04065-6)
Supplement: Supplementary file 2 — Additional file 2: Fig. S1. BUSCO assessment of unigene assembly. Fig. S2. The root samples were respectively collected at 0 h (CN), 6 h (IN), 12 h (KN) and 24 h (LN) after GXS16 inoculation, and the KEGG pathway enrichment of DEGs in the comparison of IN vs CN, KN vs IN, and LN vs KN. [file 12870_2023_4065_MOESM2_ESM.pptx]

## Slide 1
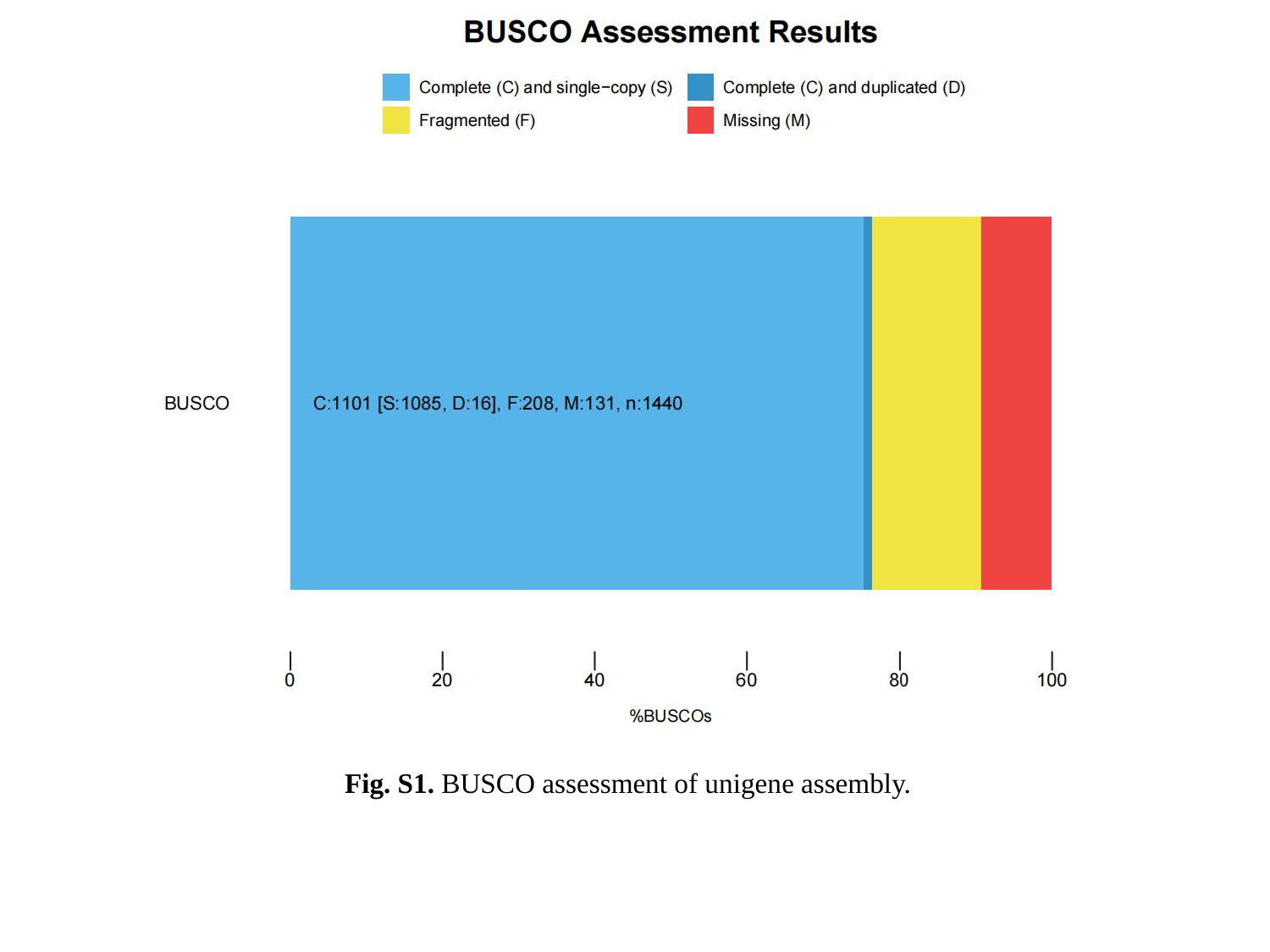

Fig. S1. BUSCO assessment of unigene assembly.

## Slide 2
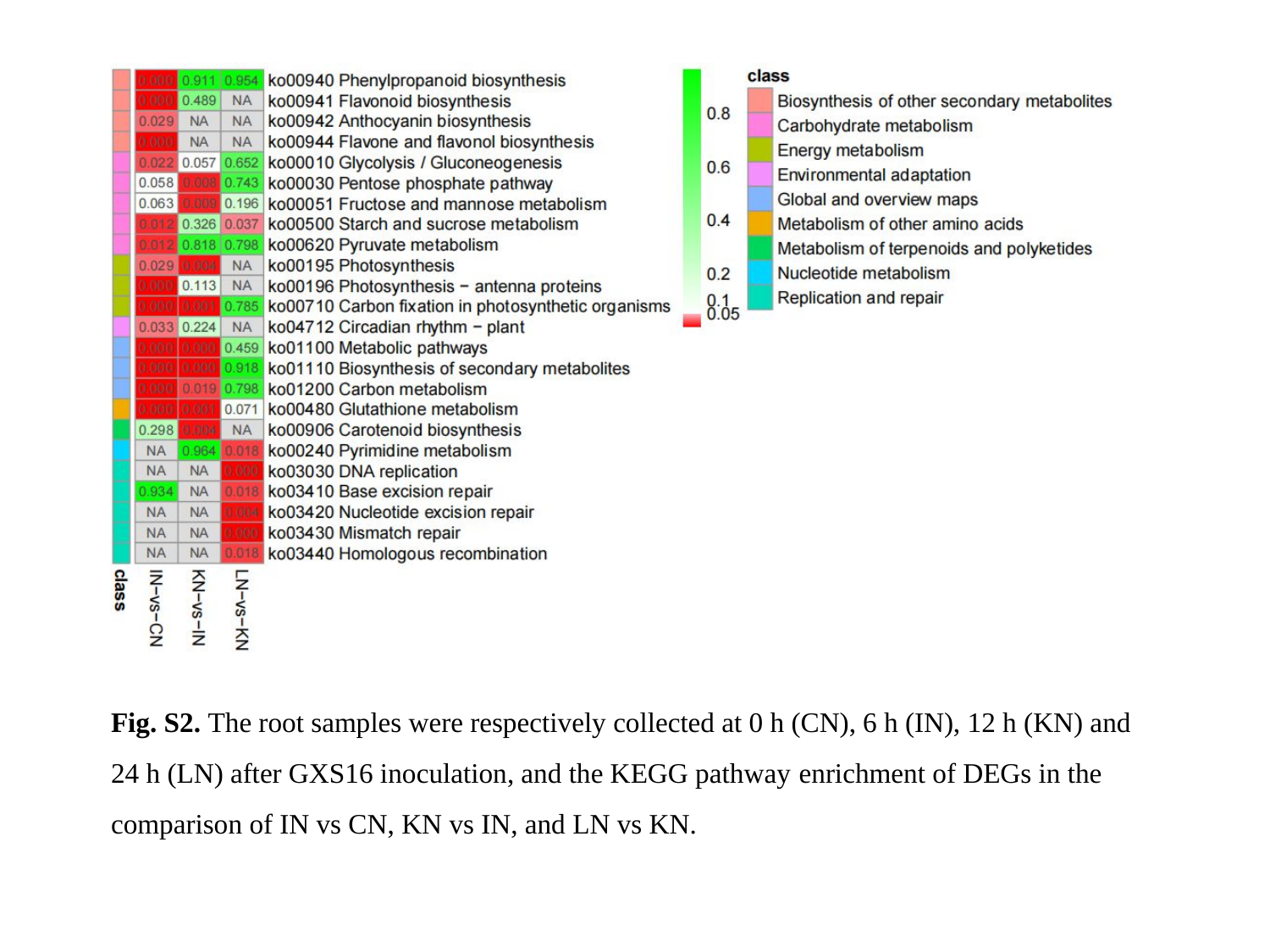

Fig. S2. The root samples were respectively collected at 0 h (CN), 6 h (IN), 12 h (KN) and 24 h (LN) after GXS16 inoculation, and the KEGG pathway enrichment of DEGs in the comparison of IN vs CN, KN vs IN, and LN vs KN.
